# Palisade structure in intact vaccinia virions

**DOI:** 10.1128/mbio.03134-23

**Published:** 2024-01-03

**Authors:** Miguel Hernandez-Gonzalez, Thomas Calcraft, Andrea Nans, Peter B. Rosenthal, Michael Way

**Affiliations:** 1 Cellular Signalling and Cytoskeletal Function Laboratory, The Francis Crick Institute, London, United Kingdom; 2 Structural Biology of Cells and Viruses Laboratory, The Francis Crick Institute, London, United Kingdom; 3 Structural Biology Science Technology Platform, The Francis Crick Institute, London, United Kingdom; 4 Department of Infectious Disease, Imperial College, London, United Kingdom; Duke University School of Medicine, Durham, North Carolina, USA

**Keywords:** vaccinia virus, poxvirus, cryo-ET, subtomogram averaging, virus structure

## Abstract

Vaccinia virus assembly in the cytoplasm of infected cells involves the formation of a biconcave viral core inside the maturing viral particle. The boundary of the core is defined by a pseudohexagonal palisade layer, composed of trimers projecting from an inner wall. To understand the assembly of this complex core architecture, we obtained a subnanometer structure of the palisade trimer by cryo-electron tomography and subtomogram averaging of purified intact virions. Using AlphaFold2 structure predictions, we determined that the palisade is formed from trimers of the proteolytically processed form of the viral protein A10. In addition, we found that each A10 protomer associates with an α-helix (residues 24–66) of A4. Cellular localization assays outside the context of infection demonstrate that the A4 N-terminus is necessary and sufficient to interact with A10. The interaction between A4 and A10 provides insights into how the palisade layer might become tightly associated with the viral membrane during virion maturation. Reconstruction of the palisade layer reveals that, despite local hexagonal ordering, the A10/A4 trimers are widely spaced, suggesting that additional components organize the lattice. This spacing would, however, allow the adoption of the characteristic biconcave shape of the viral core. Finally, we also found that the palisade incorporates multiple copies of a hexameric portal structure. We suggest that these portals are formed by E6, a viral protein that is essential for virion assembly and required to release viral mRNA from the core early in infection.

**IMPORTANCE:**

Poxviruses such as variola virus (smallpox) and monkeypox cause diseases in humans. Other poxviruses, including vaccinia and modified vaccinia Ankara, are used as vaccine vectors. Given their importance, a greater structural understanding of poxvirus virions is needed. We now performed cryo-electron tomography of purified intact vaccinia virions to study the structure of the palisade, a protein lattice that defines the viral core boundary. We identified the main viral proteins that form the palisade and their interaction surfaces and provided new insights into the organization of the viral core.

## INTRODUCTION

Poxviruses are a family of large DNA viruses that include the human pathogens variola virus—the causative agent of smallpox—and monkeypox, as well as vaccinia, which is the most studied family member. Poxviruses replicate and assemble in the cytoplasm of infected cells (1). Vaccinia virus assembly is a complex process that involves the initial assembly of spherical immature virions (IVs) (2–6). Subsequent proteolytic processing of the viral proteins A3, A10, and L4 by the protease I7 and the formation of disulfide bonds trigger the maturation of IVs into infectious intracellular mature virions (IMVs) (7–10). This maturation involves a dramatic structural reorganization of IV into brick-shaped IMV with a distinct internal core containing the genome (3, 11, 12). The core boundary is defined by a capsid-like structure with trimers projecting from an inner wall in a pseudohexagonal arrangement, which gives the appearance of a palisade (11, 13, 14). During maturation, the viral membrane becomes tightly associated with the palisade by an unknown mechanism.

Previous studies, including mass spectrometry analyses, indicate that IMVs contain more than 80 viral proteins, with ~50 being found in the viral core (3, 15, 16). Among them, A3, A4, A10, and F17 are the most abundant IMV proteins, constituting ~40% of the protein weight of the virus, which suggests that they are major structural components (16). F17 is the main component of the two lateral bodies located in two cavities between the viral membrane and the biconcave core (15, 17). A3 appears to reside inside the core and not on the palisade surface as it only becomes accessible to immunogold labeling when purified viral cores are disrupted by 3 M NaCl (18). In contrast, A4 and A10, which are essential for virion assembly (19, 20), are accessible on the surface of intact viral cores suggesting that they are palisade components. In addition, A4 has been suggested to link the viral membrane to the palisade as it is present in the membrane fraction after detergent treatment of purified virions (21). Moreover, A10 and A4 form a stable complex that can be cross-linked in purified IMVs (22, 23). Recent observations suggest that three A10/A4 heterodimers are consistent with the mass and shape of a single palisade trimer, based on a low-resolution subtomogram-averaged map of the palisade and AlphaFold2 structural predictions (11). Altogether, current evidence suggests that A10 and/or A4 form the trimeric units that constitute the palisade in intact virions.

Here, we performed electron cryo-tomography (cryo-ET) of intact IMVs and applied subtomogram averaging (STA) to obtain a more detailed molecular understanding of the virion core. Our analysis demonstrates that the palisade trimer is formed from proteolytically processed A10, as recently described for *in vitro* purified cores (24, 25). In contrast to these studies, our map also reveals that A10 associates with an additional density corresponding to a long alpha helix of A4. Localization assays in uninfected cells demonstrate that this A4 α-helix is necessary and sufficient for the interaction with A10. We also find that the palisade layer contains multiple copies of a hexameric portal structure, which we suggest is formed by the E6 viral protein.

## RESULTS AND DISCUSSION

### Cryo-ET of intact IMV reveals trimers of A10–A4 heterodimers form the palisade

**Fig 1:**
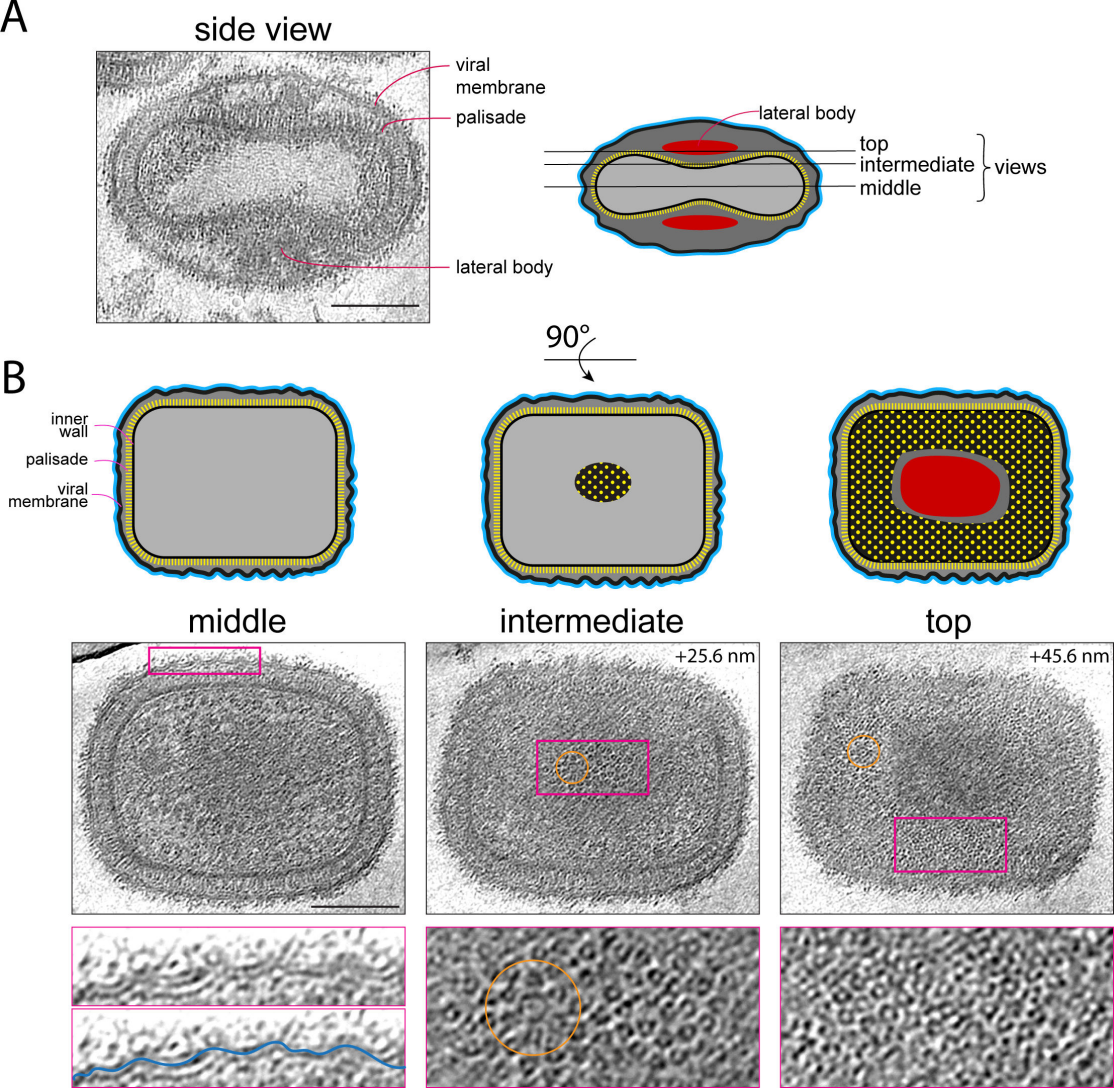
Cryo-ET of purified vaccinia virions. (A) The left shows a middle section of a tomogram showing a side view of a purified vaccinia virion, to highlight the lateral bodies located in two concavities between the viral membrane and the viral core. Scale bar = 100 nm. The right shows a schematic representation of the virion together with the tomogram positions of the three views shown in B. (**B)** The top row shows schematic representations of the broadest view of virions (90 degrees to the images shown in A). Below the schematics the corresponding middle, intermediate and top tomogram sections of a representative vaccinia virion are shown together with regions magnified three times (magenta boxes). The middle view shows the corrugated viral membrane. The intermediate view shows the surface of the palisade beneath the lateral body, demonstrating that the palisade is continuous in this region. The top view shows the palisade in the rest of the core surface, highlighting its continuity. The orange circles highlight portal complexes in the intermediate and top tomogram sections. Scale bars = 100 nm.

**Fig 2:**
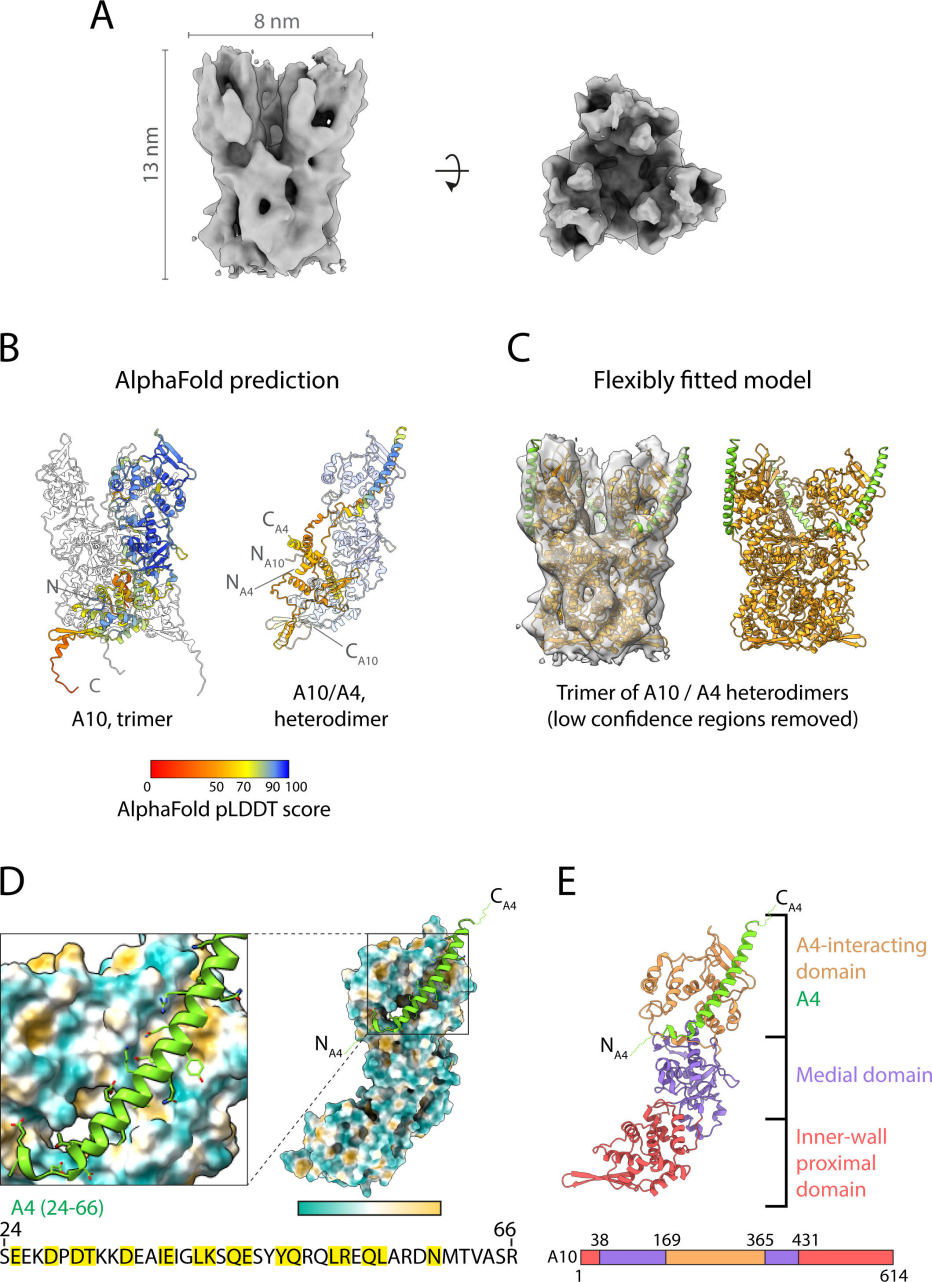
Palisade trimer structure and composition. (A) Subtomogram averaging map of the palisade trimer at 9.7 Å resolution viewed from the side (left) and top (right). Densities within 6 Å of the palisade trimer are shown for clarity. (**B)** AlphaFold2 predictions for the proteolytically processed A10 trimer (left) and A10/A4 heterodimer (right). Residues are colored by the AlphaFold2 pLDDT confidence score according to the color key. Only residues 24–66 of A4 are predicted with the highest confidence in the model. The positions of the N- and C-termini of A4 and A10 in the model are indicated. (**C)** A10 trimer together with the long A4 alpha helix (residues 24–66) flexibly fitted into the palisade map (left) after the removal of low-confidence regions in the AlphaFold2 models (residues 595–614 of A10 not included). The final model of the A10/A4 trimers after flexible fitting is shown (right). (**D)** A view of A4 residues 24–66 (green cartoon) sitting in the binding groove of A10 (surface colored by hydrophobicity according to the key, orange hydrophobic, blue hydrophilic). The protein sequence of A4 (residues 24–66) is shown, with residues contacting A10 highlighted in yellow. (**E)** Domain architecture of one protomer of proteolytically processed A10 together with the A4 alpha helix. At the bottom, linear representation of A10, with residues corresponding to domains colored as in the ribbon diagram above.

We recorded tilt series and reconstructed electron cryo-tomograms of purified infectious IMVs isolated from infected HeLa cells. The tomograms reveal that virions are structurally uniform with dimensions of ~347 × 260 × 240 nm (Fig. 1; Fig. S1). Importantly, the architecture of purified virions is identical to those imaged inside infected cells by cryo-ET (11). At the acquired magnification and defocus conditions, both leaflets of the viral membrane are discernible, whereas the inner wall of the core beneath the palisade appears as a densely packed layer that lacks membrane features (Fig. 1). The structural features of the palisade are also better resolved than in our previous study on infected cells and confirm that the palisade layer fully covers the core, including its two biconcave surfaces (Fig. 1B). To obtain a higher-resolution structure of the palisade, we conducted STA. We obtained a palisade map at a subnanometer resolution, which reveals a trimeric structure as a single conformer. The trimer is ~13 nm in height and 8 nm in diameter (Fig. 2A; Fig. S2; Tables S1 and S2) and consists of a closely packed cylindrical base that sits on top of the inner core wall, with separated lobes projecting toward the viral membrane. No structural features of the inner wall were resolved during the averaging of palisade trimers indicating that it does not share the symmetry of the palisade lattice.

Based on previous evidence indicating that A10 and/or A4 constitute the palisade layer, we compared our palisade trimer map to an AlphaFold2 prediction for the structure of A10 imposing a trimeric arrangement. As proteolytic processing of A10 occurs during IV-to-IMV maturation (8, 19, 26), we used the predicted model for the processed form of the protein (residues 1–614) (Fig. 2B; Fig. S3A). In addition, we generated a model for the full-length molecule (residues 1–891) (Fig. S3A and B). We found that most of the density in our palisade map is unambiguously explained by a model of the processed N-terminal fragment 1 of A10 (Fig. 2C). Ordered densities that would account for the C-terminal fragments 2 and 3 were not identified, suggesting that only fragment 1 of A10 is a structural component of the palisade trimer. Finally, the C-terminus of A10 fragment 1 (595–614), which is predicted with low confidence and not included in our model, is situated in a position where it could potentially interact with the inner wall (Fig. 2B). While the trimer of processed A10 accounts for most of our STA map, an elongated density (~60 Å in length) on the exterior surface of each A10 molecule remained unexplained (Fig. 2C). An AlphaFold2 model of an A10/A4 heterodimer revealed that an alpha helix of A4 (residues 24–66) located in a groove in the A10 model predictably accounts for the unexplained density (Fig. 2B through D). This region is the only part of A4 predicted with high confidence in the A10/A4 heterodimer model (Fig. S3C). Our maps, therefore, indicate that the palisade consists of an A10 trimer in which each protomer binds a long alpha helix located near the N-terminus of A4, where the N-terminus of this helix points toward the inner wall and the C-terminus points toward the viral membrane. Finally, additional unassigned densities also appear at the apex of the trimer.

Within the trimer, each A10 monomer consists of three regions: the A4-interacting domain (residues 169–364), which forms the projecting lobes at the apex of the trimer and mediates the interaction with A4 (Fig. 2D); the medial domain (residues 38–168 and 365–431), which encloses a cavity in the center of the trimer base; and the inner wall-proximal domain (residues 1–37 and 432–614), which forms the base of the trimer and interacts with the inner core wall (Fig. 2E; Fig. S3A). In our A10 model, inter-protomer interactions occur at the medial domain (residues 96–120) and the inner wall-proximal domain. In the latter case, the N-terminus (residues 1–12) packs between the opposing two protomers, interacting with residues 8–29 and 482–486. Additional interactions occur between a helix of the inner wall-interacting domain (residues 471–482) and the medial domain (residues 414–418 and 386–393) of an adjacent protomer. In total, each inter-protomer interface consists of approximately 2,285 Å^2^ of the buried surface area. In addition, the A4 helix binding in the groove on A10 buries 1,220 Å^2^ of the surface area (Fig. 2D).

### A4 interacts with A10 via its long alpha helix in the absence of additional viral proteins

**Fig 3:**
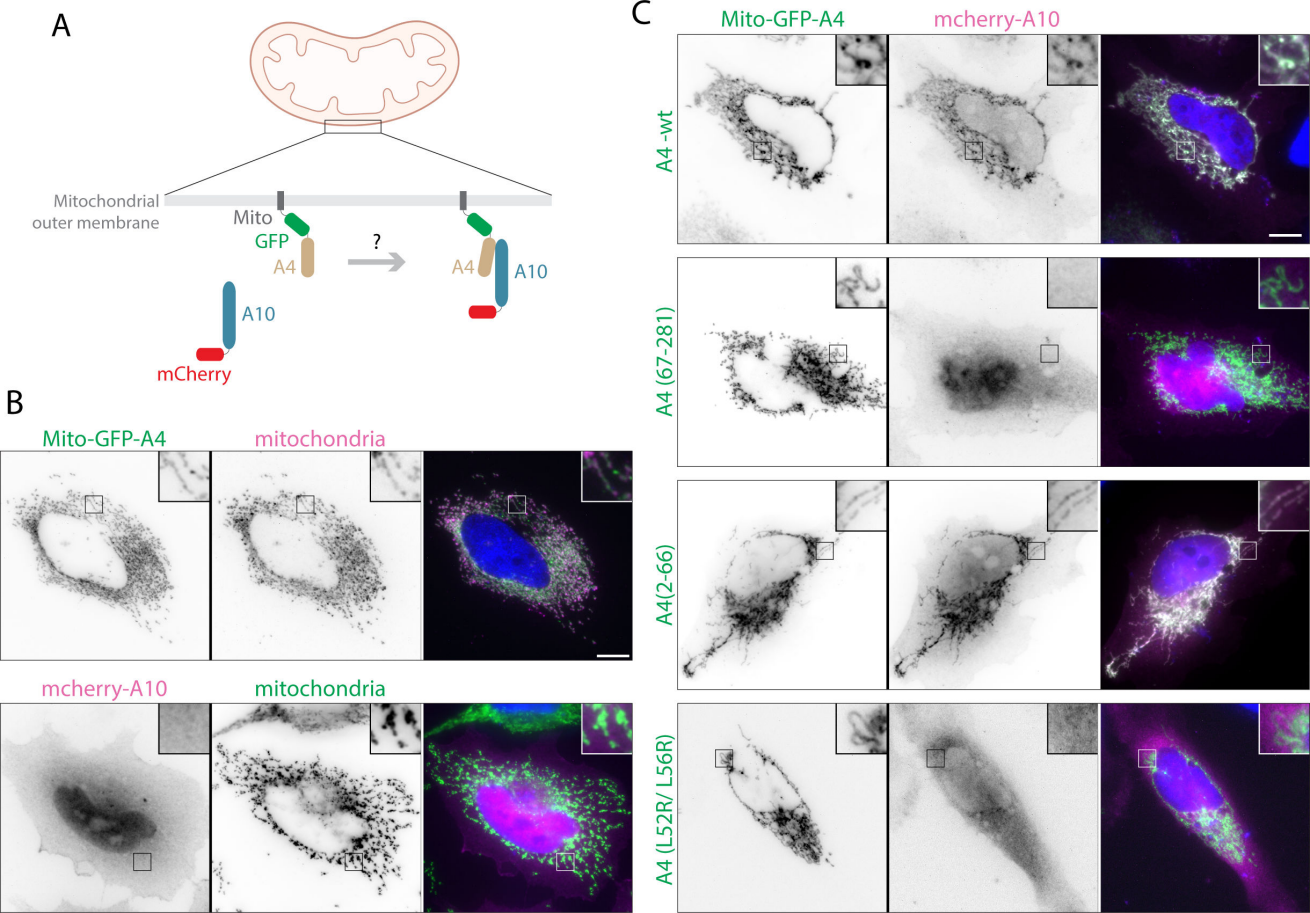
A10 is recruited to A4-decorated mitochondria in uninfected cells. (A) Schematic showing the re-localization assay used to study the interaction between A10 and A4. GFP-A4 anchored to the mitochondrial outer membrane via the Mito targeting sequence recruits cytosolic mCherry-A10 to mitochondria if there is an interaction between the two proteins. (**B)** Top row shows the colocalization of Mito-GFP-A4 with mitochondria labeled with Tom20 (magenta) in HeLa cells. The bottom row shows that mCherry-A10 is diffuse in the cytoplasm and the nucleus and not recruited to mitochondria labeled with Tom20 (green) in the absence of Mito-GFP-A4. (**C)** Representative fluorescence images of cells expressing mCherry-A10 and the indicated versions of Mito-GFP-A4. A4 and A4 (2–66) were able to recruit mCherry-A10 to mitochondria in all imaged cells, in three independent experiments. In contrast, mCherry-A10 remained diffuse in cells expressing A4 (67–281) or A4 (L52R/L56R) in all cells. Merged images also show the 4,6-diamidino-2-phenylindole (DAPI) stain nuclei in blue. Scale bars = 10 µm.

To confirm the predicted interaction of the alpha helix of A4 (residues 24–66) with A10 from our structural analysis, we performed mitochondrial recruitment assays in non-infected cells. GFP-A4, fused to a mitochondrial anchor (Mito) corresponding to the transmembrane domain of the outer mitochondrial membrane protein Tom70p (27, 28), becomes anchored on mitochondria when expressed in uninfected cells (Fig. 3A and B). We found that Mito-GFP-A4 recruits mCherry-A10 to mitochondria outside the context of infection, confirming their interaction (Fig. 3B and C). In addition, in infected cells, mCherry-A10 gets incorporated into viral particles (Fig. S4A), demonstrating that it is functional when tagged. However, mCherry-A10 was not recruited to mitochondria, remaining diffuse in the cytoplasm when a truncated version of Mito-GFP-A4 lacking residues 1–66 was used instead of wild-type protein (Fig. 3C; Fig. S4B). Furthermore, residues 2–66 of A4 were sufficient to recruit A10 to mitochondria (Fig. 3C; Fig. S4C). To confirm our findings, we sought to disrupt the interaction of the long A4 alpha helix (residues 24–66) with A10 by mutating L52 and L56, two hydrophobic residues tightly packed into the hydrophobic groove and interacting with A10 in our model. Indeed, substitutions L52R and L56R in full-length A4 abolished its ability to recruit A10 to mitochondria (Fig. 3C; Fig S4D). In conclusion, the N-terminal region of A4 (up to residue 66) is necessary and sufficient for its interaction with A10 in the absence of additional viral proteins and requires residues at the binding interface of our heterodimer model (Fig. 2D).

### Organization of the palisade layer

**Fig 4:**
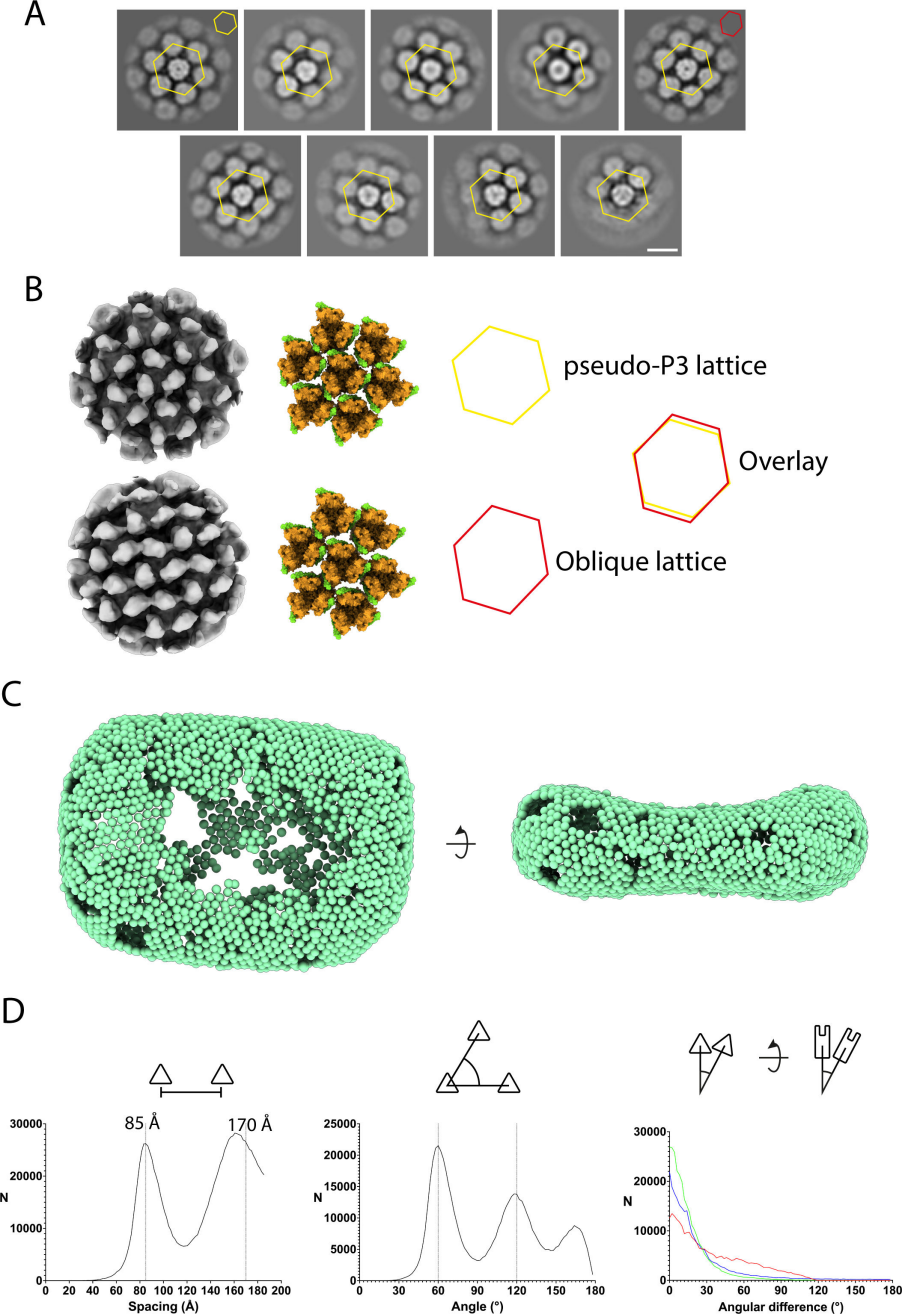
Organization of the palisade layer. (A) Gallery of central XY slices through classes from subtomogram averaging, indicating variability of the local palisade arrangement. A regular hexagon (yellow) is overlaid on each class, centered on the central trimer. Agreement with (top left) and deviation from hexagonal ordering can be observed in the positions of the centers of trimers neighboring the central one. The yellow and red hexagons in the top left and right class images, respectively, relate to the maps shown in B. Scale bar = 10 nm. (**B)** The 3D maps of two selected classes indicated in A shown as isosurfaces (left) illustrate the variability of the palisade lattice. Docking of the palisade trimer model into the two maps shows the relative position of A10 and A4 in the lattice. Yellow and red hexagons indicate regular and deformed hexagonal arrangements, respectively. (**C)** Backplotting of palisade trimer positions into the tomogram shows well-ordered and poorly ordered regions. Extended gaps are due to trimer positions not included in the final average map. (**D)** Measurements between neighboring trimers are depicted schematically above each plot. Left, histogram of inter-trimer spacings within a cut-off distance of 187 Å. The 85 Å primary spacing and the 170° harmonic are marked by dashed lines. Middle, histogram of angles between a central trimer and two neighbors within a cut-off distance of 93.5 Å. Angles at 60° and 120° are marked by dashed lines. Right, histogram of angular differences in the relative in-plane and out-of-plane dispositions of palisade trimers within a distance cut-off of 93.5 Å. The relatively strong skew of the out-of-plane differences (rlnAngleTilt, green; rlnAnglePsi, blue) indicates a tendency toward local flatness at 0°. The in-plane angular difference (rlnAngleRot, red) is more broadly distributed indicating an imperfect ordering of neighboring trimer azimuthal orientations but still with a maximum at 0° indicating hexagonal ordering.

Subtomogram averaging of the palisade trimer shows a local hexagonal ordering of neighboring trimers including classes where the hexagonal ordering is distorted (Fig. 4A). Fitting of the A10/A4 trimer model into the map shows that the palisade trimers are typically too distant to interact. This suggests that A10 interactions do not directly determine the organization of the palisade layer. However, in the most distorted lattice class, adjacent trimers make contacts involving regions of both A4 and A10 (Fig. 4A and B).

Identifying the location in virus tomograms of the individual palisade trimer subtomograms that contributed to the STA maps (“backplotting”) allowed us to visualize the larger-scale organization of the palisade layer. The palisade layer exhibits extended areas of hexagonal lattice, which are separated by less ordered regions (Fig. 4C). Analysis of spacings, angles, and relative orientation differences of nearest trimer subtomograms across the data set showed that the palisade arrangement exhibits a continuous variability deviating from a perfect hexagonal lattice (Fig. 4D). The first nearest-neighbor spacing peak is at 85 Å, while the second peak at 160 Å falls short of the 170 Å harmonic predicted for a perfect P3 lattice, consistent with regions of compression of the lattice. Angles between A10 trimers and their surrounding neighbors show peaks at 60° and 120° associated with the hexagonal arrangement, but with substantial variability. Relative in-plane angular differences between neighboring trimers in the plane of the core surface show rotational variation in how one trimer is posed relative to its neighbor. This variation is consistent with a lack of substantial direct contacts between A10 trimers. In the arrangement seen in subtomogram averaging maps (Fig. 4) or for trimers separated by the average observed spacing, the trimers show restricted rotational freedom due to clashes between the A4-interacting domains of neighboring trimers. Thus, trimers are subject to steric constraints within the palisade layer, which may explain features of the observed lattice organization. We estimate that 2,500–3,000 palisade trimers are required to constitute a viral core. The continuous inner wall beneath the palisade layer is thin and must be flexible to accommodate the curvature of the core.

The A4 helix binding sites at the periphery of A10 are close to each other at an approximate threefold axis between neighboring trimers, suggesting that A4 oligomers could cross-link A10 trimers and influence the organization of the palisade. Interestingly, AlphaFold2 predicts that A4 can trimerize near its C-terminus (230–281), which is supported by a coiled-coil prediction (residues 229–258) (Fig. S3A). It is possible that A4 trimerization could influence the organization of the palisade. However, no additional densities corresponding to an A4 trimerization domain were found in our palisade map. Furthermore, *in vitro* purified cores, which are not reported to include the A4 density seen in our STA maps, have a palisade layer in the absence of a viral membrane (24, 25). Altogether, this suggests that the palisade integrity might not require A4 and could be determined by interactions of A10 with the inner wall that remain to be described in detail (24, 25). Rather, A4 may act as a linker between the palisade and the viral membrane as previously suggested by Cudmore et al. (21). Such a tethering function for A4 would explain how the viral membrane adapts to the shape of the core during the IV-to-IMV maturation (11). The low confidence of the central region of A4 in AlphaFold predictions suggests residues 65–230 are unstructured and flexible, which would enable A4 to extend from the palisade layer to the viral membrane with or without lateral bodies. Additionally, we conclude that the wide spacing of palisade trimers allows the thin, flexible inner wall to adopt the biconcave shape of the core, although palisade trimers may also constrain or modify this flexibility such as when inter-trimer contacts occur. Additional minor protein components associated with the palisade may also contribute to its function. For example, the core component L3 is the target of TRIM5α, a viral restriction factor that, in other viruses, recognizes ordered or hexagonal lattices of similar dimensions to the palisade (29, 30).

### The palisade incorporates hexameric portal complexes

**Fig 5:**
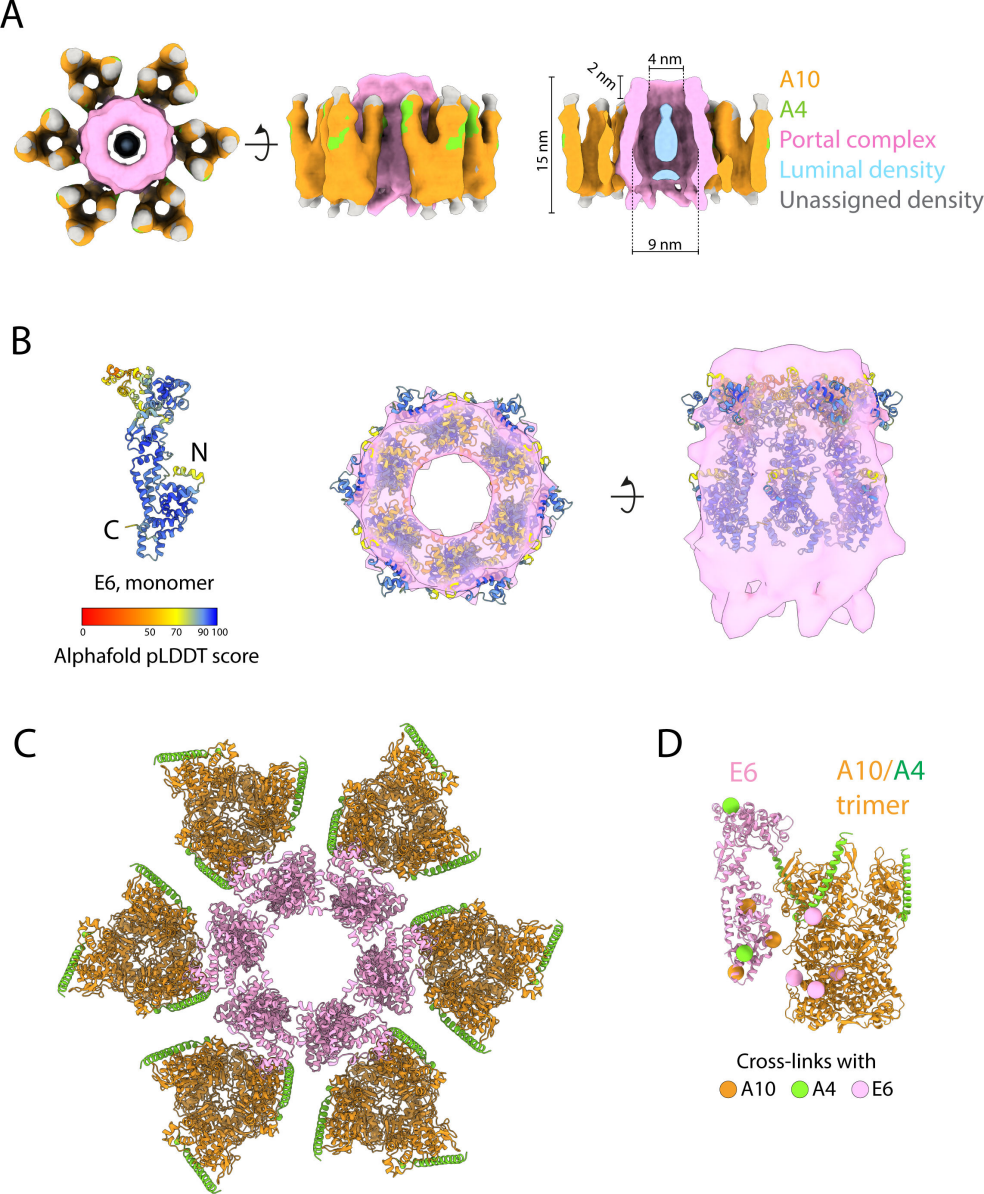
The hexameric portal complex of the palisade. (A) Subtomogram averaging map at 30.7 Å resolution of the portal complex within intact virions. The densities have been segmented and colored according to the key on the right. (**B)** Left, AlphaFold2 prediction of a vaccinia E6 monomer, colored by the pLDDT score as indicated. Middle, docking of six copies of E6 into the portal complex density segmented from the map in A, viewed from the top and side. (C) A top view of the docked atomic models of E6 and the A10/A4 trimers. (**D)** Side view of single E6 together with an A10/A4 trimer taken from the portal complex model in C showing the positions of inter-molecular cross-links identified by Mirzakhanyan and Gershon (23).

Previous observations have shown the existence of pore structures in the core of intact virions (13) as well as *in vitro* purified cores (18, 24, 25). In our IMV tomograms, we also found irregularly distributed pores within the palisade layer (Fig. 1B and 5A). These structures have a density in the center of each pore (Fig. 1B). A low-resolution STA map reveals that these pores have a hexameric architecture and contact six palisade trimers at their periphery (Fig. 5A). We refer to these hexameric pores as portal complexes as they are likely involved in the release of mRNA and/or viral DNA from the core early during infection in a similar manner to herpesviruses and bacteriophages (31–33).

From our STA map, we found that the portal complexes have a height of 15 nm, protruding 2 nm above the palisade trimers (Fig. 5A). The lumen of the portal has a maximum diameter of ~9 nm in its inner chamber and a minimum diameter of 4 nm in the protruding region. The density observed in the lumen of the portal does not extend into the protruding region. Given the similarities with other viral portals, this luminal density might correspond to nucleic acid. In addition, the ~13 nm outer diameter of the portals interrupts the lattice spacing of the palisade. The surrounding palisade trimers make lateral contacts with the portal via their A4-interacting and medial domains (Fig. 5A). These portal-associated A10/A4 trimers have an additional density on top of their A4-interacting domains that is absent in palisade trimers without an adjoining portal complex (Fig. 5A).

Based on the contouring of our STA map of the portal complex, the estimated mass of the hexameric portal is around 300–600 kDa, implying that the monomeric unit would be in the range of 50–100 kDa. Previous studies have identified approximately 50 viral proteins within vaccinia cores (3, 16). Most of these proteins are too small (<40 kDa) to form a portal complex by themselves. Among the possible viral protein candidates to form the portal complex, we focused our attention on E6, a 67-kDa protein present in cores (34). First, cross-linking data from purified IMV reveals connections between E6 and A10 as well as A4 that are compatible with an organization where palisade trimers make lateral contacts with E6 (23). Second, E6 is essential for virion assembly (34, 35). Lastly, a temperature-sensitive E6 P226L mutant does not impair virus assembly at the restrictive temperature, but the resulting cores are transcriptionally inactive and do not release RNA *in vitro* (36). Taken together, these data suggest that E6 may be a major constituent of the portal complex. The AlphaFold2 prediction of the E6 monomer is an elongated molecule of length ~112 Å and width ~43 Å (Fig. 5B). When docked into the portal complex, the resulting model of an E6 hexamer is compatible with the size and shape of the portal, supporting the notion that E6, in close association with A4/A10, is the major viral protein forming the portal complex (Fig. 5C), although additional components might explain extra densities. Moreover, the interacting surfaces between E6 and A4/A10 in the docked model contain the residues that have previously been cross-linked (23) (Fig. 5D).

**Fig 6:**
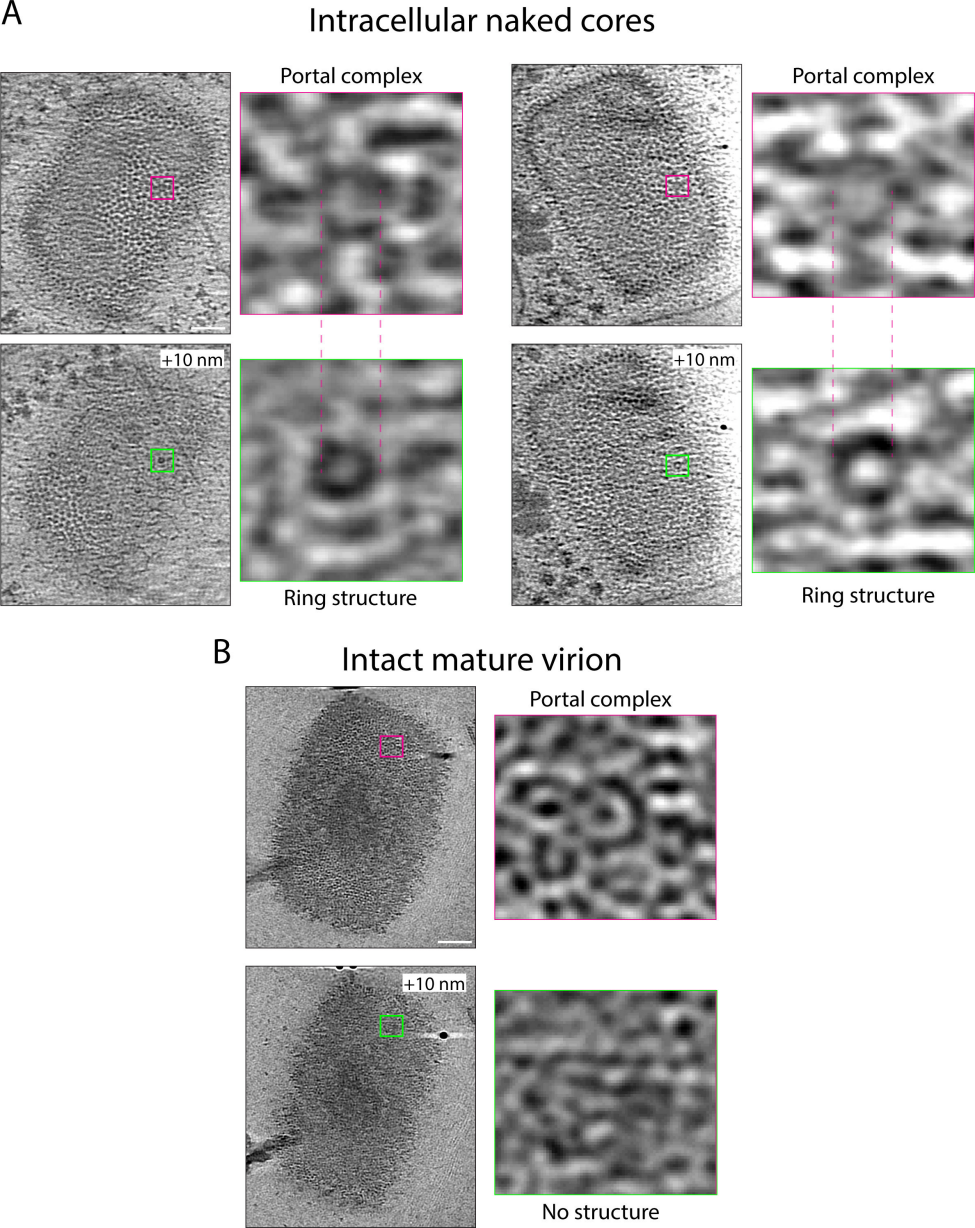
Intracellular cores have aligned portal complexes and rings. (A) Tomogram sections of two intracellular cores from our previous study (11). The top image is at the surface of the palisade, highlighting a portal complex with a magenta square. The lower image is a +10 nm section of the same tomogram, showing that the ring structure (green box) we previously described is situated on top of the portal complex. The images to the right of the full viral core images show a close-up view (10 times magnified) of the portals and rings in the highlighted boxes. (**B)** Equivalent views of a purified virion, showing a portal complex and, 10 nm above, the absence of a ring structure. Scale bars = 50 nm.

Our previous cryo-ET analysis of infected cells described the presence of ring structures on the palisade surface of cytoplasmic naked cores (11). These rings were not visible on IMV cores suggesting that they might correspond to the hexameric rings formed by the viral D5 helicase, which is recruited to naked cores early in infection to promote the release of the viral genome (i.e., uncoating) (37, 38). As proposed by Datler et al. (25), it is possible that the rings we described are related to the portal complexes found in purified IMV. However, the portals only protrude ~2 nm above the palisade in contrast to the ~17 nm protrusion of the rings. Furthermore, the portal complex cannot be composed of D5 as this protein is not found in purified virions (23, 37). Therefore, although similar in dimensions and abundance, we believe that the hexameric portals constitute a different structure from the rings. After re-examination of our previous tomograms of intracellular cores in the cytoplasm of infected cells (11), we have found portal complexes within the palisade ~10 nm below the ring structures, suggesting that these two different structures might be coupled (Fig. 6A; Movie S1). Moreover, we were unable to detect rings above portals in purified virions (Fig. 6B). D5 is essential for genome release into the cytoplasm, which occurs after the release of early viral mRNAs, one of which encodes D5 (37). An attractive possibility is that portal complexes are initially used for early mRNA release, and the subsequent docking and helicase activity of a hexameric D5 ring repurposes the portal complex to promote genome release.

We have shown that the main constituent of the palisade layer, the A10 trimer, has a key role in organizing the virus architecture by associating with the core inner wall, incorporating portals during assembly, and interacting with A4, which may act as a linker to the viral membrane (21). How A4 would contribute to the association of the viral membrane with the palisade during IV-to-IMV maturation remains to be established. Future work will also be required to confirm if E6 is a major component of the portal complexes and examine their possible function in virion assembly, as well as mRNA extrusion and D5-mediated genome release early in infection.

## MATERIALS AND METHODS

### Purification of intact vaccinia virions

Monolayers of HeLa cells were infected with vaccinia [Western Reserve (WR) A36-YdF strain (39), multiplicity of infection (MOI) = 0.05] for 3 days. Cells were scraped in phosphate-buffered saline (PBS) and centrifuged at 3,000 × *g* for 5 min at 4°C. The cells were resuspended in PBS, centrifuged again, and resuspended in virus buffer (2 mM MgCl_2_, 10 mM Tris-HCl, pH 9). To release the intracellular virions, cells were lysed by 20 strokes of a Dounce homogenizer. The resulting lysate was centrifuged for 5 min at 3,000 × *g* at 4°C, and the viral supernatant was loaded on top of an 8 mL 35% sucrose cushion in a Beckman SV40 ultracentrifuge tube. Virus buffer was added to make up a total volume of 30 mL. After centrifugation at 24,000 rpm for 30 min at 4°C in a Beckman Optima L-100 XP ultracentrifuge, the pellet, containing purified vaccinia virions, was resuspended in virus buffer and stored at either −80°C or −20°C. The virus titer was determined by infecting monolayers of BS-C-1 cells with serial dilutions of the purified virus.

### Vitrification

Intact virions were diluted 2- or 10-fold in PBS and mixed with colloidal gold particles (10 nm diameter). A 4 µL sample of the diluted virus was pipetted onto glow discharged (40 s at 45 mA) Quantifoil R2/2 copper grids of 300 mesh and blotted for 3 or 4 s with a relative force of −10, 95% relative humidity, and 22°C, using the Vitrobot Mark IV System.

### Electron cryo-tomography, image processing, and analyses

Cryo-ET data collection was carried out on a 300 kV Titan Krios microscope equipped with a Falcon 4 camera behind a Selectris energy filter operated with a slit width of 10 eV. The pixel size was 1.56 Å at the specimen plane. The applied defocus ranged between −2 and −5 µm over the course of the data collection. Tilt series were recorded using a dose-symmetric tilt scheme with a range of ±60° in 3° increments. The total dose of 98.6 e^−^/Å^2^ was fractionated over the 41 tilt micrographs, resulting in a dose of 2.4 e^−^/Å^2^ per tilt. Movies were motion-corrected using Relion’s implementation of the MotionCor2 algorithm (40) in 3 × 3 patches, regrouping the 396 camera frames in the EER format to six MRC frames per micrograph. CTF parameters were then estimated using Gctf (41). Tilt series were aligned, and tomograms were reconstructed using IMOD (42). Tomograms were denoised using Topaz for visualization (43).

### Subtomogram averaging

Core surfaces were segmented in Dynamo (44), and oversampled positions on the surfaces were exported into Relion 4.0 for subtomogram averaging (45). Subtomograms were initially extracted at fourfold binning (6.24 Å/pixel) in a 64-pixel box, before the initial classification against a model generated using Relion’s initial model function, with local searches ±30° around the initial rlnAngleTilt and rlnAnglePsi Euler angles assigned by Dynamo. Once particles had aligned to the palisade lattice (initial model), particles from poor classes and overlapping particles within a 50 Å distance cutoff were removed, and particles were reextracted in a 48-pixel box at fourfold binning followed by additional classification. Refinement then proceeded with only local orientation searches and without imposition of symmetry. After convergence at binning level 4, particles were reextracted at a bin2 (3.12 Å/pixel) box size of 96 pixels, and further classifications and refinements applied a mask around the central palisade trimer while imposing C3 symmetry.

The final map of the palisade trimer was reconstructed from the tilt series images without binning (1.56 Å/pixel) in a 192-pixel box with C3 symmetry imposed. The resolution was calculated using a soft mask over the central palisade trimer. The class averages in Fig. 4A were generated by the asymmetric reclassification of particles into 12 classes at fourfold binning in a 64-pixel box after the convergence of the final unbinned refinement, with a local search of the out-of-plane rotations and free rotation in the plane of the core surface. Asymmetric classifications at all stages of processing supported our interpretation of a single trimer conformation and full occupancy of the A4 binding sites.

Portal complexes were manually selected, pre-oriented in ArtiaX (46), and extracted at fourfold binning in Relion in a 64-pixel box. The initial model was generated by the reference-free classification with one class and no applied symmetry. Subsequent refinement enforced C6 symmetry and employed local searches of the out-of-plane rotations with free rotation in the plane of the core surface. The final map was reconstructed from the tilt series images without binning in a 256-pixel box with C6 symmetry imposed. The resolution was calculated using a spherical mask.

Subtomogram particle sets and alignments were visualized and curated by placing aligned positions and orientations back in the tomogram reference frame using ArtiaX. Nearest-neighbor analysis of inter-trimer spacings, lattice angles, and relative angular differences, similar to that in (47), was carried out using a custom Python script.

### Modeling

The models of homotrimeric A10 and heterodimeric A10/A4 were predicted by AlphaFold2 in the multimer mode (48). The A10 trimer was fitted as a rigid body into the subtomogram averaging density using ChimeraX (49), before applying a further rigid fitting operation to only the A4-interacting domain to optimize fit to density. Alignment of the A10 portion of the A10/A4 heterodimer model to one protomer of the A10 trimer placed the predicted A4 helix residues 24–66 into the unmodeled tubular density. The C-terminal A10 residues 595–614 were removed as they could not be assigned to any observed density feature. The model was then subjected to restrained molecular dynamics flexible fitting in ISOLDE (50), applying restraints to all inter-atomic distances ≤8 Å to maintain the predicted protein fold. Servalcat/Refmac5 was used for a final refinement in Fourier space to calculate the model map FSC (51). Validation was carried out in Phenix (52).

The model of monomeric E6 was predicted by AlphaFold2 (53). It was then fitted as a rigid body into the portal complex density map in ChimeraX, along with the surrounding palisade trimer models. The coiled-coil prediction was carried out using the Waggawagga server for consensus prediction (54).

### Molecular biology

A4 was amplified from the DNA of the Western Reserve vaccinia strain using the primers MHG107 (5′- TACAAGGGCGGCCGCGACTTCTTTAACAAGTTCTCACAG-3′) and MHG108 (5′- TGCAGGAATTCTTACTTTTGGAATCGTTCAAAACCTTTG-3′), and after digestion with NotI/EcoRI, the PCR product was cloned in frame with GFP into the pEL-GFP vector (55). GFP-A4 was subsequently amplified from this plasmid using the primers MHG119 (5′-ATCCACCGGTCGCCACCATGAGCAAGGGCGAGGAGCTG-3′) and MHG120 (5′-ATATCTCGAGTTACTTTTGGAATCGTTCAAAACCTTTG-3′) and digested with AgeI and XhoI. The Mito tag was obtained from pMito-mCherry-FRB (Addgene #59352) by HindIII/AgeI digestion. These two DNA fragments were then ligated into the XhoI and Hind III sites of 3xHA-TurboID-NLS_pCDNA3 (Addgene #107171). The fidelity of the final CMV promoter-driven expression vector Mito-GFP-A4, which has a linker of MDELYKGGR connecting GFP and A4, was confirmed by sequencing. The coding sequences corresponding to residues 2–66 and 67–281 were cloned in place of full-length A4 to generate Mito-GFP-A4 (2–66) or Mito-GFP-A4 (67–281) expression vectors, respectively. Mito-GFP-A4 (L52R L56R) was made by cloning A4 (L52R L56R) in place of A4 Mito-GFP-A4 (67–281) plasmid. A4 (L52R L56R) was generated by site-directed mutagenesis with primers MHG114 (5′-GTATTATCAAAGACAGAGGCGAGAACAAAGAGCTAGAGATAATATG-3′) and MHG115 (5′-CATATTATCTCTAGCTCTTTGTTCTCGCCTCTGTCTTTGATAATAC-3′) of the GFP-A4 plasmid. To make the mCherry-A10 plasmid, A10 was amplified with primers MHG121 (5′-ATATGCTAGCATGATGCCTATTAAGTCAATAGTTACTC-3′) and MHG122 (5′-ATATCTCGAGTTATTCATCATCAAAAGAGACAGAG-3′) and digested with NheI and XhoI. mCherry was amplified from mCherry-hALIX (Addgene #21504) with primers MHG129 (5′-AAGCTTGCGGCCGCCACCATGGCCTCCTCCGAGGAC-3′) and MHG130 (5′-ATATGCTAGCGGCGCCGGTGGAGTGGCG-3′) and digested with NotI and NheI. Finally, these two DNA fragments were then ligated into the NotI/XhoI sites of 3xHA-TurboID-NLS_pCDNA3 (Addgene #107171).

### Transient transfection, fluorescence imaging, Western blotting, and infection

HeLa cells were seeded on fibronectin-coated coverslips and transfected with 1 µg of the corresponding plasmid/s using FUGENE as described by the manufacturer (Promega). Twenty hours after transfection, the cells were fixed with 4% paraformaldehyde in PBS for 15 min, permeabilized with 0.1% Triton X-100 for 5 min, and incubated in blocking buffer [10 mM MES (pH 6.1), 150 mM NaCl, 5 mM EGTA, 5 mM MgCl_2_, and 5 mM glucose] containing 2% (vol/vol) fetal calf serum and 1% (wt/vol) BSA for 30 min prior to the addition of DAPI for 5 min, before mounting the coverslips using Mowiol. When mitochondria were labeled, prior to DAPI staining, coverslips were incubated for 1 h with a mouse monoclonal antibody against Tom20 (1:500 dilution in blocking buffer, Sc-17764, Santa Cruz Biotechnology) followed by a 1 h incubation with either Alexa Fluor 647 or Alexa Fluor 488 anti-mouse secondary antibodies (1:1,000 dilution, A21235 or A21202, respectively, Thermo Fisher). Coverslips were imaged on a Zeiss Axioplan2 microscope equipped with a 63×/1.4 NA Plan-Achromat objective and a Photometrics Cool Snap HQ camera. The microscope was controlled with MetaMorph 7.8.13.0 software. The experiment was repeated three times, and in each case, 10 transfected cells were imaged. Fiji was used for image analysis. Parallel to coverslip preparation, HeLa cells were also grown in six-well plates and transfected with the same plasmids. After 20 h of transfection, cells were scraped and resuspended in 1 mL PBS and pelleted by centrifugation at 0.5 × *g* for 5 min. Pellets were incubated with 25 µL lysis buffer [PBS, 1% SDS, protease and phosphatase inhibitor cocktail (5872, Cell Signalling Technology) and 0.8 µL benzonase (70746, Millipore)] at room temperature for 5 min. Then, 1× NuPAGE LDS buffer (NP0007, Invitrogen) and 100 mM DTT were added and heated for 10 min at 70°C. Finally, 10 µL was loaded onto 4%–12% SDS-polyacrylamide gels, transferred to nitrocellulose, immunoblotted with GFP mouse monoclonal (1:1,000 dilution, Cancer Research, UK) or anti-vinculin (1:2,000 dilution, V4505, Sigma) antibodies, and developed after the incubation with peroxidase-conjugated goat anti-mouse antibody (1:10,000, 115-036-146, Jackson ImmunoResearch).

HeLa cells, grown on fibronectin-coated coverslips, were infected with the vaccinia Western Reserve strain at a MOI of 1. Cells were subsequently transfected with a plasmid expressing mCherry-A10 at 1 h post-infection. Finally, cells were fixed with 4% paraformaldehyde in PBS 8 h post-infection. Coverslips were processed for immunofluorescence as above, and after permeabilization, mature virions were labeled with an A27 antibody [1:2,000; Rodriguez et al. (56)] and incubated with Alexa Fluor 488 anti-mouse secondary antibody.

### Map, model, and tomogram deposition

EM maps from tomography (EMD-18916) and subtomogram averaging (portal complex: EMD-18917 and palisade trimer: EMD-18918) have been deposited in the Electron Microscopy Data Bank. The fitted atomic model of the A10/A4 palisade trimer has been deposited in the Protein Data Bank with the ID 8R5I.

## SUPPLEMENTAL MATERIAL

Fig. S1 mbio.03134-23-s0001.pdf Dimensions of vaccinia virions.

Fig. S2 mbio.03134-23-s0002.pdf Fourier shell correlation plots for the palisade map and model.

Fig. S3 mbio.03134-23-s0003.pdf Alphafold2 A10 and A4 structural predictions.

Fig. S4 mbio.03134-23-s0004.pdf mcherry-A10 expression in infected cells and protein integrity of Mito-GFP-A4 mutant versions.

Movie legend mbio.03134-23-s0005.docx Movie S1 legend.

Supplemental tables mbio.03134-23-s0006.docx Tables S1 and S2.

Movie S1 mbio.03134-23-s0007.mp4 Intracellular cores have aligned portal complexes and rings.

ASM does not own the copyrights to Supplemental Material that may be linked to, or accessed through, an article. The authors have granted ASM a non-exclusive, world-wide license to publish the Supplemental Material files. Please contact the corresponding author directly for reuse.
