# Supplementary material for: Palisade structure in intact vaccinia virions
Source: mBio. 2024 Jan 3;15(2):e03134-23. doi: 10.1128/mbio.03134-23 (PMC10865856; doi:10.1128/mbio.03134-23)
Supplement: Fig. S1 — Dimensions of vaccinia virions. [file mbio.03134-23-s0001.pdf]

A

| | long axis | intermediate axis | minor axis |
|---|---|---|---|
| average (nm) | 346.76 | 259.58 | 240.35 |
| SEM | 1.46 | 1.38 | 5.94 |
| n | 77 | 66 | 11 |

B

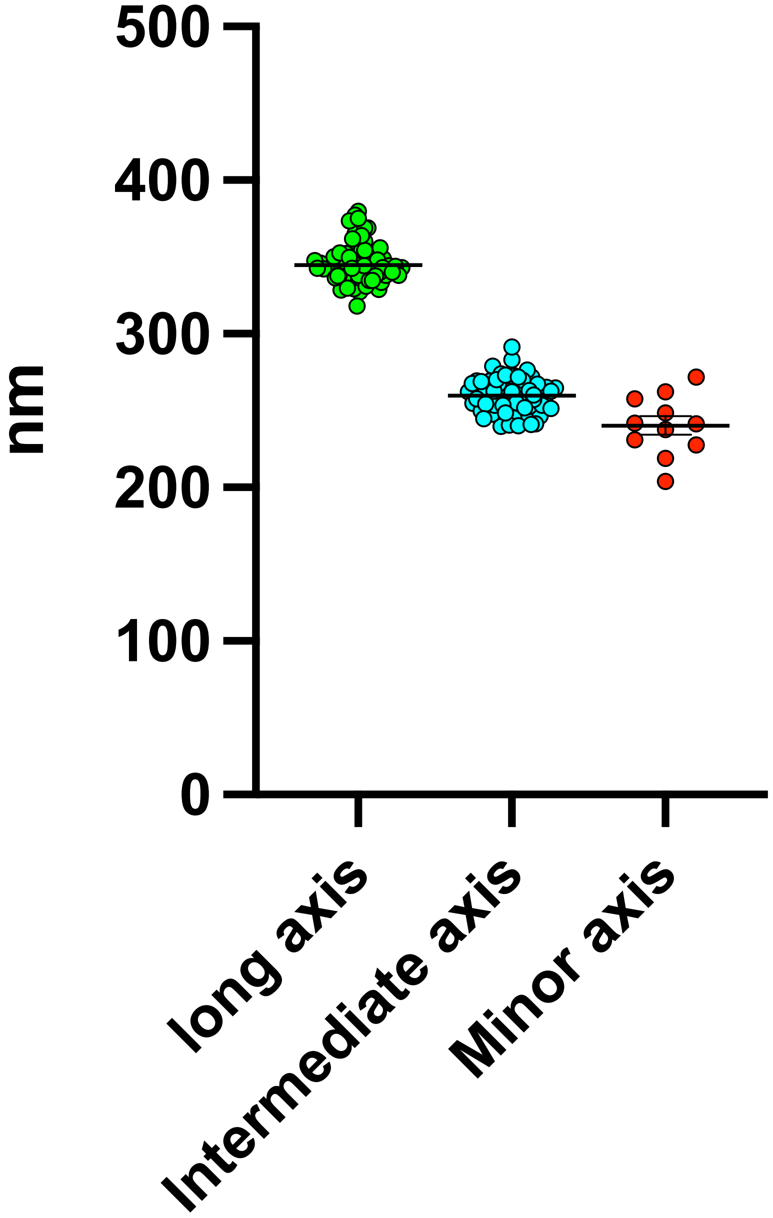


**Figure S1. Dimensions of vaccinia virions**
**A.** The table shows the dimensions of the three main axes of the virions together with the standard error of the mean and the number of virions analysed. There are less measurements for the minor axis as side views perpendicular to the electron beam are infrequent. **B.** The graph shows the individuals values for the length of the three axes of vaccinia virions.
