# Supplementary material for: Palisade structure in intact vaccinia virions
Source: mBio. 2024 Jan 3;15(2):e03134-23. doi: 10.1128/mbio.03134-23 (PMC10865856; doi:10.1128/mbio.03134-23)
Supplement: Fig. S2 — Fourier shell correlation plots for the palisade map and model. [file mbio.03134-23-s0002.pdf]

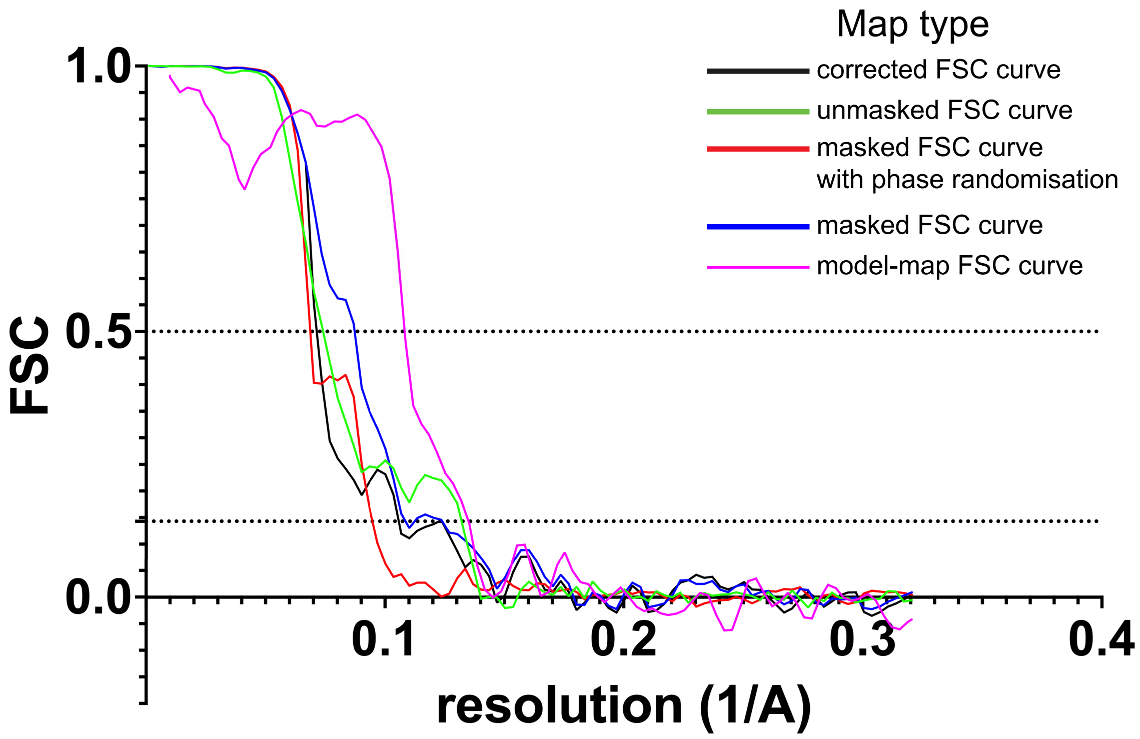

Figure S2. Fourier shell correlation plots for the palisade map and model

Curves are given for the corrected, unmasked, masked and masked with phase randomisations half-map FSCs, as well as the model-map FSC.
