# Supplementary material for: Palisade structure in intact vaccinia virions
Source: mBio. 2024 Jan 3;15(2):e03134-23. doi: 10.1128/mbio.03134-23 (PMC10865856; doi:10.1128/mbio.03134-23)
Supplement: Fig. S3 — Alphafold2 A10 and A4 structural predictions. [file mbio.03134-23-s0003.pdf]

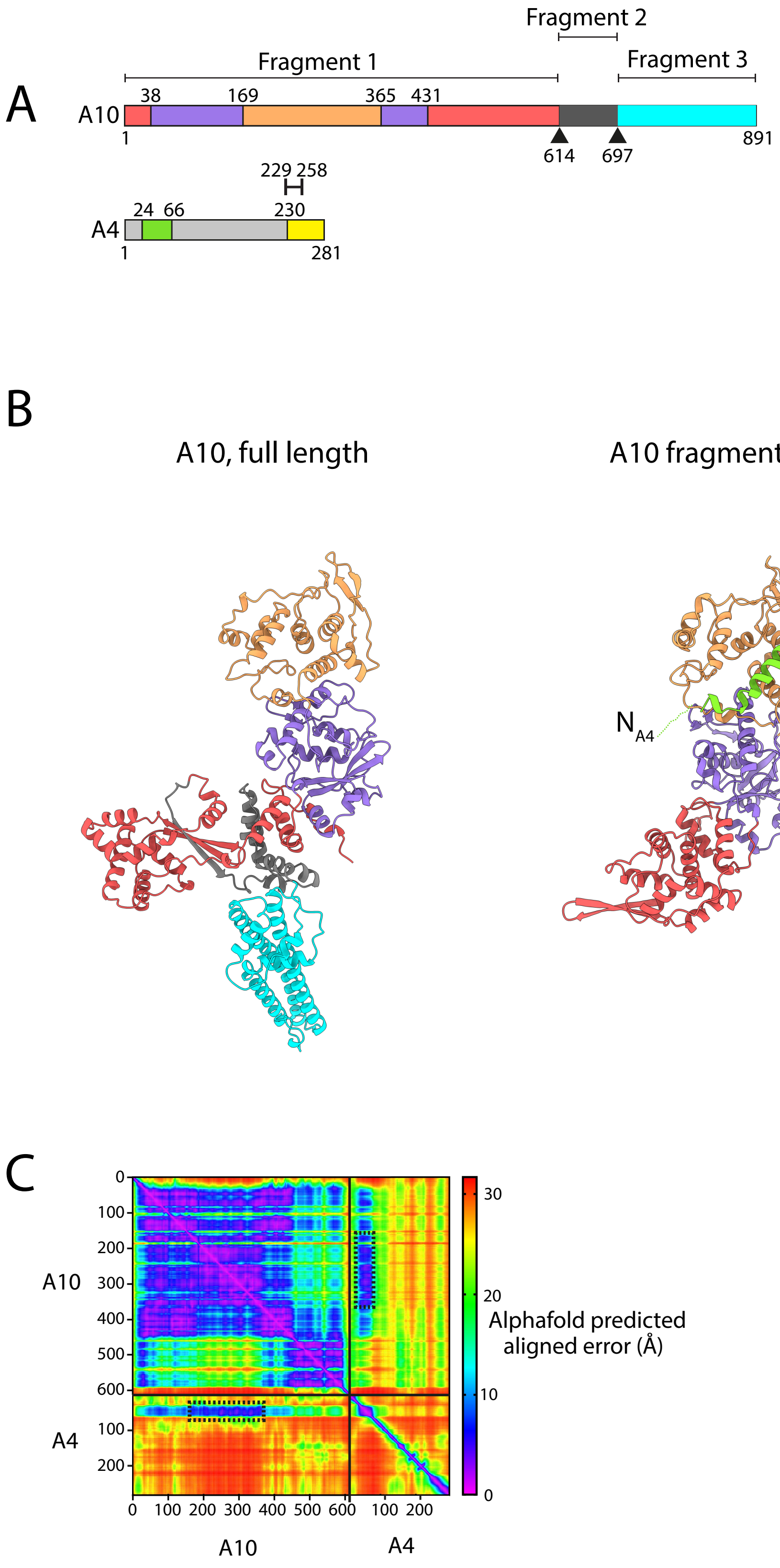

Figure S3. Alphafold2 A10 and A4 structural predictions
A. A schematic of unprocessed A10 showing the residues and regions that form the three A10-fragment-1 domains described in figure 2 (orange, A4-interacting domain; purple, medial domain; red, inner-wall proximal domain) as well as fragment 2 (grey) and 3 (blue). Black arrows indicate the proteolytic cleavage sites in A10 after the indicated residues. The A4 schematic shows the position of the A10 interacting alpha helix (green), the predicted trimerisation domain (yellow) from Alphafold2 as well as the predicted C-terminal coiled coil region (229-258). **B.** Alphafold2 model of full length A10, coloured as in A, next to the model shown in Figure 2E, for comparison. **C.** Alphafold2 predicted aligned error (PAE) plot for the A10/A4 heterodimer. The dashed boxes indicate regions of low PAE (high confidence) corresponding to the interaction between the A4 helix and the A4-interacting domain of A10.
