# Supplementary material for: Palisade structure in intact vaccinia virions
Source: mBio. 2024 Jan 3;15(2):e03134-23. doi: 10.1128/mbio.03134-23 (PMC10865856; doi:10.1128/mbio.03134-23)
Supplement: Fig. S4 — mcherry-A10 expression in infected cells and protein integrity of Mito-GFP-A4 mutant versions. [file mbio.03134-23-s0004.pdf]

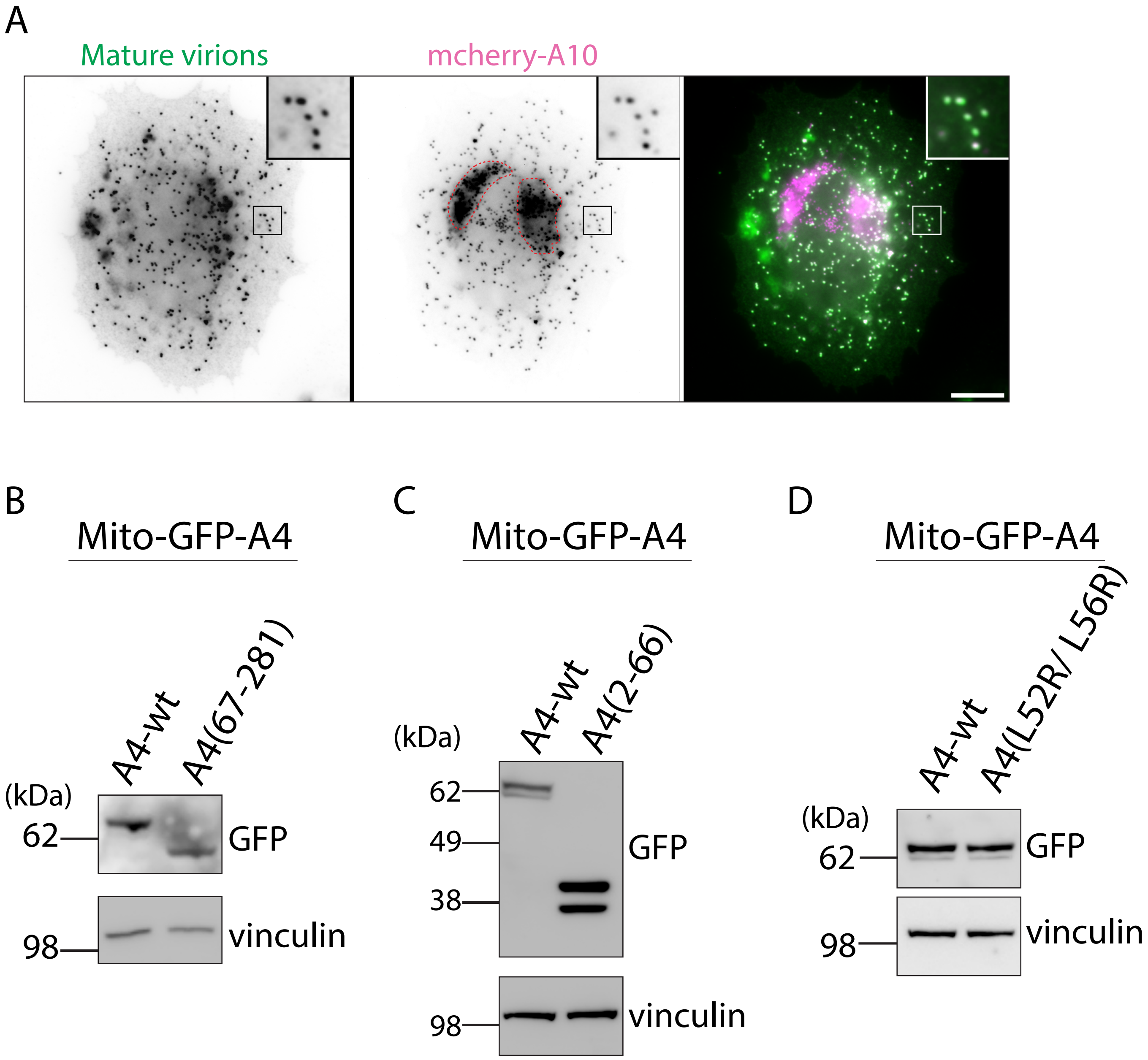

Figure S4. mcherry-A10 expression in infected cells and protein integrity of Mito-GFP-A4 mutant versions.

A. Immunofluorescence image showing that mcherry-A10 (magenta) localises to mature virions labelled with an A27 antibody in a HeLa cell infected with WR for 8 hours. Dotted lines label A10-containing immature virions in the viral factories. Inserts are 3-times magnified. Scalebar = 10 µm **B, C, D.** Immunoblots of cell lysates expressing wild type Mito-GFP-A4 and its indicated mutant blotted with antibodies against GFP and vinculin, as a loading control.
