# Supplementary material for: Palisade structure in intact vaccinia virions
Source: mBio. 2024 Jan 3;15(2):e03134-23. doi: 10.1128/mbio.03134-23 (PMC10865856; doi:10.1128/mbio.03134-23)
Supplement: Movie legend — Movie S1 legend. [file mbio.03134-23-s0005.docx]

**Movie S1. Intracellular cores have aligned portal complexes and rings.** Movie showing a tomogram of an intracellular naked core with portal complexes (magenta box) beneath ring structures (green box). The speed of the movie is reduced in frames showing portal complexes and ring structures, for better appreciation.
