# Supplementary material for: Palisade structure in intact vaccinia virions
Source: mBio. 2024 Jan 3;15(2):e03134-23. doi: 10.1128/mbio.03134-23 (PMC10865856; doi:10.1128/mbio.03134-23)
Supplement: Supplemental tables — Tables S1 and S2. [file mbio.03134-23-s0006.docx]

**Table S1. Cryo-ET data acquisition and processing.**

| **Microscope** | **FEI Titan Krios** | |
| --- | --- | --- |
| Acceleration voltage (keV) | 300 | |
| Detector | Thermo Fisher Falcon 4 | |
| Energy filter | Thermo Fisher Selectris, slit width 10eV | |
| Defocus range (µm) | -2 to -5 | |
| Magnification | 81,000x | |
| Pixel size (Å) | 1.56 | |
| Tilt scheme | Dose symmetric, ±60° in 3° increments with grouping of 2 | |
| Total number of tilt series | 100 | |
| Number of micrographs per tilt series | 41 | |
| Dose rate (e^-^/Å^2^/sec) | 2.2 | |
| Dose rate (e^-^/pixel/sec) | 4.5 | |
| Exposure time per micrograph (sec) | 1.29 | |
| Dose per micrograph (e^-^/Å^2^) | 2.4 | |
| EER frames per micrograph | 396 | |
| Total dose per tilt series (e^-^/Å^2^) | 98.6 | |
| **Data processing** | **Palisade trimer** | **Portal complex** |
| Initial particle number | 623174 | 86 |
| Final particle number | 123492 | 86 |
| Point group symmetry | C3 | C6 |
| Map resolution (Å) at FSC=0.143 | 9.7 | 30.7 |

**Table S2. Model statistics for the palisade trimer model**

| **Model statistics** |  |
| --- | --- |
| Model resolution at FSC=0.5 (Å) | 9.21 |
| Clash score | 8.53 |
| Rotamer outliers (%) | 3.34 |
| Ramachandran outliers (%) | 0.16 |
| MolProbity score | 1.95 |
| Cβ deviations (%) | 0.11 |
| Bond RMSDs |  |
| Bond lengths (Å) | 0.008 |
| Bond angles (°) | 1.546 |
